# Retraction: Large Pleural Lipoma Manifesting With Chronic Shoulder Pain

**DOI:** 10.7759/cureus.r125

**Published:** 2024-01-25

**Authors:** Aljawharah A Alotaibi, Fawaz S Albaqami, Asim K Almushayqih, Ghadeer N Alotaibi, Najd F Aldakkan, Jumanah M Muammar, Ahmed A Alsulaimani, Khlood I Alrowathi, Abdulwahab A Almansour, Saad S Alrushud, Alaa A Aldeibani, Amal H Allohaibi, Basmah M Aljohani, Ahmed Aljohani, Faisal Al-Hawaj

**Affiliations:** 1 Medicine, King Abdulaziz University, Jeddah, SAU; 2 Medicine, Qassim University, Qassim, SAU; 3 Medicine, Dar Al-Uloom University, Riyadh, SAU; 4 Medicine, Ibn Sina National College for Medical Studies, Jeddah, SAU; 5 Medicine, King Saud University, Riyadh, SAU; 6 Medicine, Imam Abdulrahman Bin Faisal University, Dammam, SAU

The Editors-in-Chief have retracted this article. Concerns were raised regarding the identity of the authors on this article. Specifically, Faisal Alhaway and Malak Shammari have stated that they were added to this article without their knowledge or approval. The identity of the other authors could also not be verified. As the appropriate authorship of this work cannot be established, the Editors-in-Chief no longer have confidence in the results and conclusions of this article.

